# Case report: Pesticide-related methemoglobinemia: Tebufenozide and indoxacarb poisoning

**DOI:** 10.3389/ftox.2025.1557990

**Published:** 2025-02-27

**Authors:** Jieru Wang, Guangcai Yu, Tianzi Jian, Baotian Kan, Wei Li, Xiangdong Jian

**Affiliations:** ^1^ Department of Critical Care Medicine, The 5th People’s Hospital of Jinan, Jinan, Shandong, China; ^2^ Department of Poisoning and Occupational Diseases, Emergency Medicine, Qilu Hospital of Shandong University, Cheeloo College of Medicine, Shandong University, Jinan, Shandong, China; ^3^ Department of Nephrology, The Affiliated Hospital of Shandong University of Traditional Chinese Medicine, Jinan, Shandong, China; ^4^ Department of Geriatric Medicine, Department of Nursing, Qilu Hospital of Shandong University, Cheeloo College of Medicine, Shandong University, Jinan, Shandong, China

**Keywords:** methemoglobinemia, tebufenozide, indoxacarb, pesticide, poisoning

## Abstract

**Background:**

Methemoglobinemia can be inherited or acquired. Acquired forms are more common due to drugs or poisonous substances that oxidize hemoglobin, and pesticide-related cases are notably rare.

**Case Presentation:**

We report a 69-year-old woman who ingested 30 mL of tebufenozide and indoxacarb and was asymptomatic for 3 h; however, the patient was admitted to the hospital after 8 h, unconscious, with tachypnea, cyanosis, and 61.9% methemoglobin. The patient was administered methylene blue, mechanically ventilated, and hemoperfused. Subsequently, the patient recovered and was discharged with no discomfort and with normal laboratory test results.

**Conclusion:**

Tebufenozide and indoxacarb may cause methemoglobinemia, leading to cyanosis, unconsciousness, and respiratory failure; therefore, they should be handled with care in clinical practice.

## 1 Introduction

Methemoglobinemia is a rare clinical condition manifesting as excessive methemoglobin (MetHb) in the blood due to the oxidation of iron from the Fe2+ to the Fe3+ state (Yu et al., 2022; McNulty et al., 2022). MetHb can reduce oxygen-binding capacity and shift the oxygen dissociation curve to the left, leading to anemia, hypoxia, and tissue damage. Additionally, the hypoxia is refractory to oxygen supplementation (McNulty et al., 2022; Shi et al., 2022). Methemoglobinemia can be congenital or acquired. Acquired methemoglobinemia are more common and are frequently caused by exogenous substances such as nitrates, nitrites, local anesthetics (e.g., benzocaine, prilocaine, and lignocaine), dapsone, and aniline (which requires metabolic activation) (Kamath et al., 2022; Belzer and Krasowski, 2024). Pesticide-related methemoglobinemia are rare. We present a case involving the ingestion of tebufenozide and indoxacarb, which illustrates the intoxication mechanism and reviews diagnostic clues and treatment options.

This study was approved by the Ethics Committee of Shandong University Qilu Hospital (KYLL-202309-028). The family of the patient provided written informed consent for participation in this study, which included a data availability statement.

## 2 Case presentation

**FIGURE 1:**
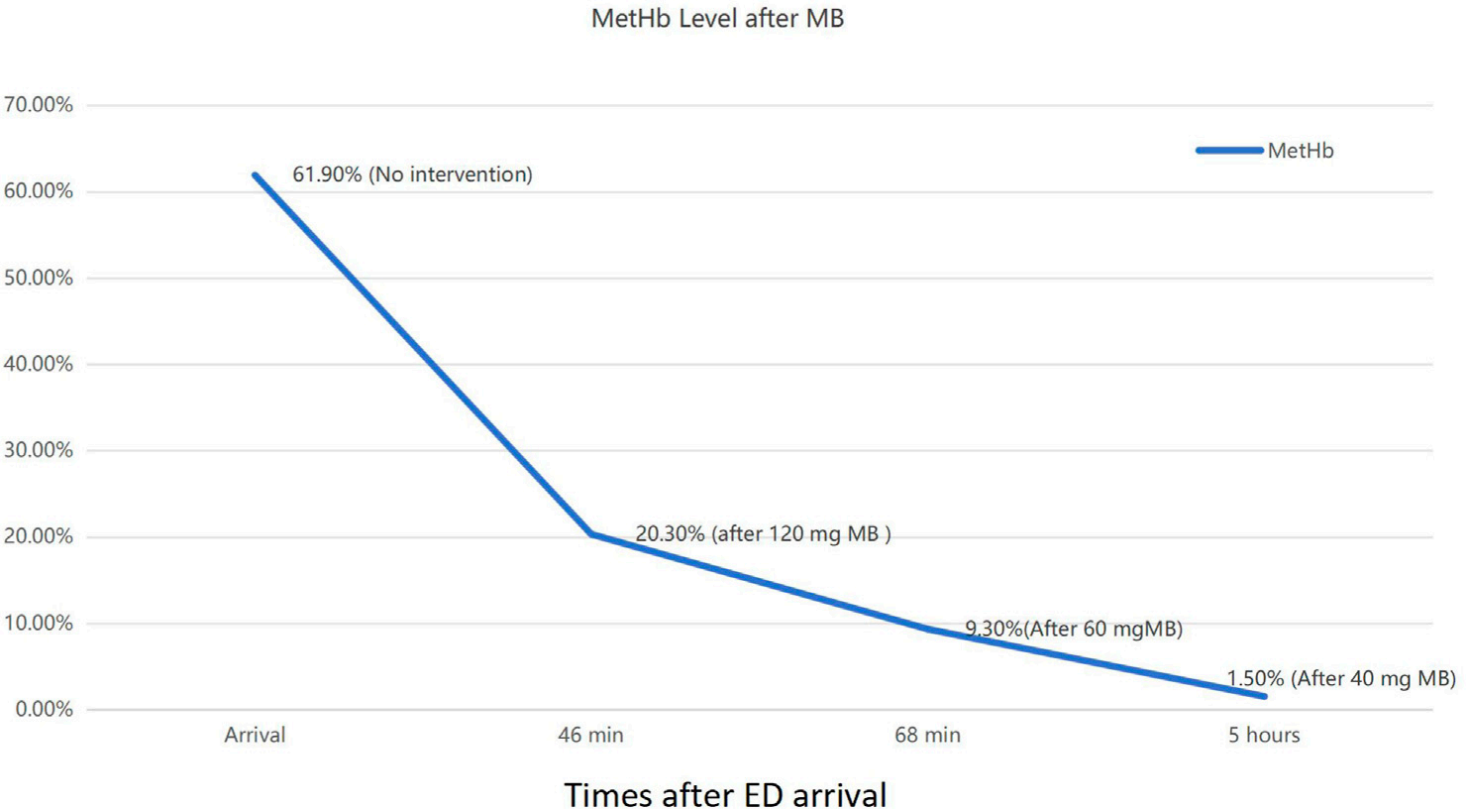
Patient methemoglobin (MetHb) levels after methylene blue (MB) administration.

A 69-year-old woman (height, 160 cm; weight, 65 kg) with a history of diabetes mellitus but no hereditary disorders was admitted at a local hospital unconscious and with cyanosis 8 h after ingesting 30 mL of an insecticide (tebufenozide and indoxacarb, W/V, 8%). The patient had no discomfort and fell asleep after taking 0.8 mg of alprazolam. The patient had no significant abnormalities 5 h after ingestion of the insecticide; however, the patient became unconscious with tachypnea and cyanosis an hour later and was admitted with a Glasgow Coma Scale score of 6 (E1V2M3), body temperature of 35.5°C, heart and respiratory rate of 63 and 28 bpm, respectively, blood pressure 179/79 mmHg, and pulse oximetry of 70%. The lips and nail beds of the patient exhibited cyanosis; however, heart and chest examination results were normal. Arterial blood gas (ABG) analysis using FiO_2_ (fraction of inspired oxygen) at 60% revealed a pH of 7.36, arterial oxygen pressure (PaO_2_) of 315 mmHg, oxyhemoglobin of 36.3%, actual bicarbonate of 28.2 mmol/L, and MetHb of 61.9% (Table 1). Gastric lavage, endotracheal intubation, mechanical ventilation, and methylene blue (MB) were administered. The MetHb level of the patient decreased from 61.9% to 20.3% and 9.3% after intravenous administration of 120 and 60 mg of MB, respectively. The patient was transferred to the Shandong University of Qilu Hospital 14 h after receiving 40 mg of MB in transit. The MetHb levels after different doses of MB are shown in Figure 1. The patient and her family had no known history of hemolysis. On admission, the vitals of the patient were stable under sedation. The ABG results FiO_2_ 40% indicated a pH of 7.47, partial oxygen pressure (PO_2_) of 241.8 mmHg, O_2_ concentration of 97.4%, and MetHb of 1.5%. Tebufenozide and indoxacarb blood levels were 0.097 μg/mL and 0.059 μg/mL, respectively. Peripheral blood cells, hepatic and renal function, myocardial enzymes, electrolytes, coagulation function, and cholinesterase levels were normal. After hemoperfusion and symptomatic treatment, the patient regained consciousness, had stable vital signs, and was disconnected from mechanical ventilation. Once the patient’s consciousness was restored, a thorough account of the poisoning was collected. The patient was discharged 4 days after insecticide ingestion with no discomfort and normal laboratory test results. Three days post-discharge, the patient underwent routine blood tests, along with liver and kidney function evaluations at a local hospital, all showing normal results. The examination 15 days after poisoning revealed no abnormalities in physical or neurological assessments. Comprehensive testing, including electrocardiogram, blood routine, urinalysis, liver and renal function, myocardial enzymes, and coagulation profile, was unremarkable.

**TABLE 1:** Arterial blood gases of the patient investigated in this study.

Inspection time	pH	pCO_2_ (mmHg)	pO_2_ (mmHg)	O_2_ Hb	HHb	MetHb	SO_2_	Lac (mmol/L)	FiO_2_
2024-5-22; 7:50	7.36	50	315	36.3%	2.3%	61.9%	94%	2.4	60%
2024-5-22; 8:26	7.40	40	277	80.7	1%	20.3%	100%	1.7	100%
2024-5-22; 9:58	7.44	37	400	87.5%	1.3%	9.3%	98.5%	1.1	40%
2024-5-22; 13:53	7.47	30.4	241.8	97.4%	0.7%	1.5%	99.3%	1.8	33%

Clinical vitals include pH, partial pressure of CO_2_ (pCO_2_), partial pressure of O_2_ (pO_2_), oxyhemoglobin (O_2_Hb), Deoxyhemoglobin (HHb), oxygen saturation (SO_2_), lactic acid (Lac), and the fraction of inspired oxygen (FiO_2_).

## 3 Discussion

Tebufenozide, an insect growth regulator that mimics ecdysone, causes premature and incomplete molting and controls lepidopteran pests (Yu et al., 2016). Tebufenozide is less toxic to vertebrates and invertebrates (LD_50_ > 5,000 mg/kg for oral uptake) than other insecticides (Addison, 1996; Abass, 2016). Potential metabolites of tebufenozide-like aromatic amines may induce methemoglobinemia; however, detoxification prevents their formation in hepatic microsomes (Abass, 2016). Therefore, although low doses of tebufenozide are not expected to cause methemoglobinemia and no cases have been reported, this association remains uncertain and requires further verification in clinical cases. Indoxacarb is a broad-spectrum non-organophosphorus oxadiazine pro-insecticide with active metabolites inhibiting voltage-gated sodium channels in insect nerve cells, impairing nerve function and leading to death (Lin et al., 2023). Indoxacarb has low mammalian toxicity, with oral absorption of approximately 60% and primary accumulation in fat tissues and red blood cells (European Food Safety Authority EFSA et al., 2018). Indoxacarb can be metabolized into active aromatic intermediates, which may lead to methemoglobinemia, hemolytic anemia, acute kidney injury, and rhabdomyolysis (Jin, 2012; Park et al., 2011; Sivanandam et al., 2024).

Methemoglobinemia severity directly correlates with MetHb levels, affecting tissue oxygen delivery. Possible symptoms include: asymptomatic, pale or gray skin, and low pulse oximetry (MetHb < 10%); cyanosis, dark brown blood, tachycardia, lightheadedness, anxiety, and confusion (MetHb ≥ 10%); fatigue, dyspnea, chest pain, dizziness, headache, and syncope (MetHb ≥ 30%); tachypnea, acidosis, dysrhythmias, delirium, seizures, and coma (MetHb ≥ 50%); and severe hypoxemia or death (MetHb ≥ 70%) (Iolascon et al., 2021; Khan et al., 2020). Other symptoms used for the diagnosis include central cyanosis (Iolascon et al., 2021), chocolate-colored arterial blood (Warren and Blackwood, 2019), an oxygen saturation gap (Das and Singh, 2024), and low pulse oximeter readings (SpO_2_) (Ward et al., 2019). These abnormalities, which are not influenced by cardiac or respiratory factors, do not improve effectively with supplemental oxygen. Notably, the saturation gap, which is the difference between PaO_2_ and SpO_2_, may increase with oxygen therapy (Das and Singh, 2024). Standard pulse oximeters cannot differentiate between bound hemoglobin forms, including dyshemoglobins, which are unsuitable for evaluating methemoglobinemia severity (Ward et al., 2019). In our case, the percentages of O_2_Hb, HHb, and MetHb do not add up to 100%, with a total exceeding 100%. We speculate that the differences observed may arise from the interference of methemoglobin with certain measurement parameters.

Treatment of methemoglobinemia should be based on the symptoms of the patient, MetHb levels, and underlying causes. MB administration is the primary treatment for symptomatic patients with MetHb levels >20% and asymptomatic patients with MetHb levels >30% (Khan et al., 2020). Before administering MB the patient’s G6PD Deficiency should be assessed. This is crucial as G6PD deficiency can influence the response to MB treatment and predispose individuals to hemolysis. MB is administered intravenously at an initial dose of 1–2 mg/kg for over >3–5 min, with a maximum dose of 5.5 mg/kg, as doses exceeding 7 mg/kg can worsen the condition (Iolascon et al., 2021). MetHb levels require repeated tests every 30 min, and if no significant decrease is observed, re-administration is necessary (Tseung et al., 2023). The patient was administered 40 mg of methylene blue, reducing the MetHb level from 9.3% to 1.5%, demonstrating the therapeutic potential of methylene blue at doses below the commonly cited range of 1–2 mg/kg body weight. Ascorbic acid can substitute MB or can be contraindicated to reduce oxidative stress and MetHb levels. There is no standardized optimal ascorbic acid dosage, and its effects are slower than those of MB. Previous case reports suggested that effective doses range from 1 to 10 g (Park et al., 2017; Park et al., 2014). N-acetylcysteine is reported to treat methemoglobinemia, but some studies have indicated that it does not reduce MetHb levels *in vitro* or in human volunteers (Dötsch et al., 2000; Tanen et al., 2000).

Timely clearance of poisons is crucial for minimizing systemic effects and enhancing patient outcomes due to their dose-dependent toxicity. Gastric lavage and bowel irrigation can remove poisons from the gastrointestinal tract before absorption by the body (Zellner et al., 2019; Carboni Bisso et al., 2020). Blood purification can remove absorbed poisons from the bloodstream and ameliorate metabolic imbalances (Saleh et al., 2022). Although indoxacarb is lipid-soluble, the concentration of indoxacarb in plasma was found to be about one-fifth of its concentration in red blood cells (Ai et al., 2024), indicating that its plasma protein binding rate is relatively low. This suggesting that hemoperfusion should not be utilized as a primary therapeutic measure for tebufenozide-indoxacarb poisoning. Blood transfusions and exchanges can restore normal hemoglobin levels; however, their effectiveness may be compromised by residual poisons. Although high-flow oxygen does not increase oxygen saturation levels, it can increase dissolved oxygen concentration, as indicated by the PO_2_, which is the form available to tissues; thus, it can partially alleviate tissue hypoxia (Lien et al., 2021). Mechanical ventilation and extracorporeal membrane oxygenation can increase dissolved oxygen levels and provide essential life support, benefiting patients with refractory methemoglobinemia (Lien et al., 2021; Zuo et al., 2023). Additionally, methemoglobinemia-related complications such as hemolytic anemia, acute kidney injury, and toxic encephalopathy require monitoring and timely intervention.

## 4 Conclusion

This study described the successful treatment of methemoglobinemia caused by ingestion of tebufenozide and indoxacarb using MB, and mechanical ventilation. Although insecticides rarely cause methemoglobinemia, this case illustrates that they must be considered as causal agents.

## Data Availability

The original contributions presented in the study are included in the article/supplementary material, further inquiries can be directed to the corresponding authors.
